# Slow Breathing Can Be Operantly Conditioned in the Rat and May Reduce Sensitivity to Experimental Stressors

**DOI:** 10.3389/fphys.2017.00854

**Published:** 2017-10-30

**Authors:** Donald J. Noble, William N. Goolsby, Sandra M. Garraway, Karmarcha K. Martin, Shawn Hochman

**Affiliations:** Department of Physiology, Emory University School of Medicine, Atlanta, GA, United States

**Keywords:** operant conditioning, slow, deep breathing, experimental stressors, animal model, physio-behavioral monitoring

## Abstract

In humans, exercises involving slowed respiratory rate (SRR) counter autonomic sympathetic bias and reduce responses to stressors, including in individuals with various degrees of autonomic dysfunction. In the rat, we examined whether operant conditioning could lead to reductions in respiratory rate (RR) and performed preliminary studies to assess whether conditioned SRR was sufficient to decrease physiological and behavioral responsiveness to stressors. RR was continuously monitored during 20 2-h sessions using whole body plethysmography. SRR conditioned, but not yoked control rats, were able to turn off aversive visual stimulation (intermittent bright light) by slowing their breathing below a preset target of 80 breaths/min. SRR conditioned rats greatly increased the incidence of breaths below the target RR over training, with average resting RR decreasing from 92 to 81 breaths/min. These effects were significant as a group and vs. yoked controls. Preliminary studies in a subset of conditioned rats revealed behavioral changes suggestive of reduced reactivity to stressful and nociceptive stimuli. In these same rats, intermittent sessions without visual reinforcement and a post-training priming stressor (acute restraint) demonstrated that conditioned rats retained reduced RR vs. controls in the absence of conditioning. In conclusion, we present the first successful attempt to operantly condition reduced RR in an animal model. Although further studies are needed to clarify the physio-behavioral concomitants of slowed breathing, the developed model may aid subsequent neurophysiological inquiries on the role of slow breathing in stress reduction.

## Introduction

An emerging view is that mind-body techniques involving slowed deep breathing lead to considerable health benefits in humans. Deep breathing exercises such as those in pranayama (“manipulation of the breath movement”) yoga have been found to improve stress-related physiological functions including autonomic imbalance, cardiopulmonary and neuroendocrine function, and mood (Brown and Gerbarg, 2005; Jerath et al., 2006; Kaushik et al., 2006; Courtney, 2009; Pramanik et al., 2009). There is evidence that slowed deep breathing increases cardiac variability by a factor of two or more (Lehrer et al., 1999; Elliott and Edmonson, 2005) and cuts self-reported depression in half (Janakiramaiah et al., 1998). This suggests volitional slowed deep breathing can reduce sympathetic tone with consequent reduction in stress (Jerath et al., 2006).

It is commonly understood that respiratory rate (RR) is heightened during stress and reduced during rest and slow wave sleep (Pappenheimer, 1977; Suess et al., 1980; Stephenson et al., 2001; Mortola, 2004; Guijt et al., 2007). Furthermore, given previously described evidence of the therapeutic benefits of slowed respiratory rate (SRR), as well as the suggestion that high RR predicts a number of negative cardiopulmonary outcomes (Fieselmann et al., 1993; Hodgetts et al., 2002), RR appears to be an important index of stress and its deleterious impact on behavior.

Despite evidence of a therapeutic benefit to SRR in disorders of autonomic imbalance (Bernardi et al., 2002; Brown and Gerbarg, 2005; Joseph et al., 2005; Jerath et al., 2006; Kaushik et al., 2006; Courtney, 2009; Pramanik et al., 2009), human studies of SRR techniques have had difficulty ruling out extraneous variables as contributors to these changes (e.g., expectancy biases developed during training). Moreover, it has proven difficult to formulate proper control groups, and very few studies have focused on isolating SRR from attentional or emotional regulatory elements (Ospina et al., 2007). Sustained SRR could be sufficient to evoke both acute (Bernardi et al., 1998; Harinath et al., 2004) and adaptive (Han et al., 1996; Pal et al., 2004) changes in the central nervous system corresponding to reduced sympathetic autonomic tone, but new approaches are needed to isolate the impact of SRR from confounding variables.

We set out to resolve this issue using behavioral conditioning procedures to reduce RR in an animal model (the rat), and then explore whether SRR leads to physio-behavioral changes consistent with reduced stress reactivity. Previous investigators have tried to control respiration using classical and operant conditioning with varying degrees of success (Ley, 1999). Classical conditioning approaches remove the volitional component involved in human SRR techniques, produce a range of respiratory responses that are not limited to changes in RR, and strongly depend on the unconditioned stimulus used to initially provoke the response (Gallego and Perruchet, 1991; van den Bergh et al., 1995; Nsegbe et al., 1997, 1998). In comparison, operant conditioning protocols have had limited success altering respiration in rats and humans (Gallego et al., 1994; Ley, 1999; Elliott and Izzo, 2006). The only successful attempt to (indirectly) operantly condition RR in the rat was a pilot study that used electrical stimulation of the medial forebrain bundle as reinforcement (Gallego et al., 1994)—and this study only included one animal.

We undertook a non-invasive approach to reduce RR and determine whether SRR conditioned rats would retain slowed respiration into the post-training period. We then performed a series of exploratory tests to investigate the impact of training on physiological and behavioral responsiveness to three experimental stressors: novelty stress (open field), restraint, and nociceptive stimuli. The studies presented herein support the fundamental proposition that long-term reductions in RR can be achieved non-invasively and non-pharmacologically in an animal model, and provide preliminary evidence linking slowed RR to anxiety/stress reduction in established behavioral tests.

## Results

**Figure 1:**
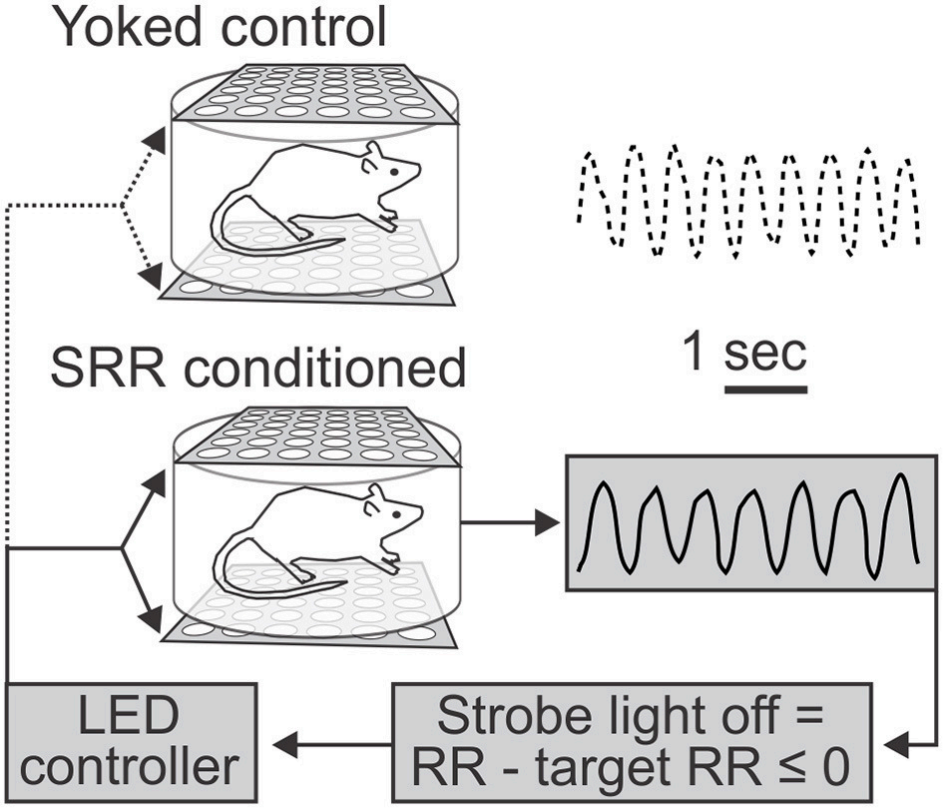
Use of light as negative reinforcement for respiratory rate feedback. Schematic depicts feedback procedure and representative respiratory traces from yoked control (**top**) and slowed respiratory rate (SRR) conditioned (**bottom**) animals over identical 5 s time intervals. Respiratory rate (RR) was continuously recorded via whole body plethysmography. For conditioning, real-time RR was compared to a predetermined target RR by a controller coupled to a transducer to trigger the light stimulus, such that the strobe light was turned off whenever current RR ≤ target RR (set to ≤ 80 breaths/min). Animals thereby experienced real-time changes in strobe illumination corresponding to their RR. This represents a closed-loop feedback control system for conditioning of SRR. In the example traces shown, the average RRs were 70.5 (conditioned) and 91.0 (yoked control) breaths/min.

**Figure 2:**
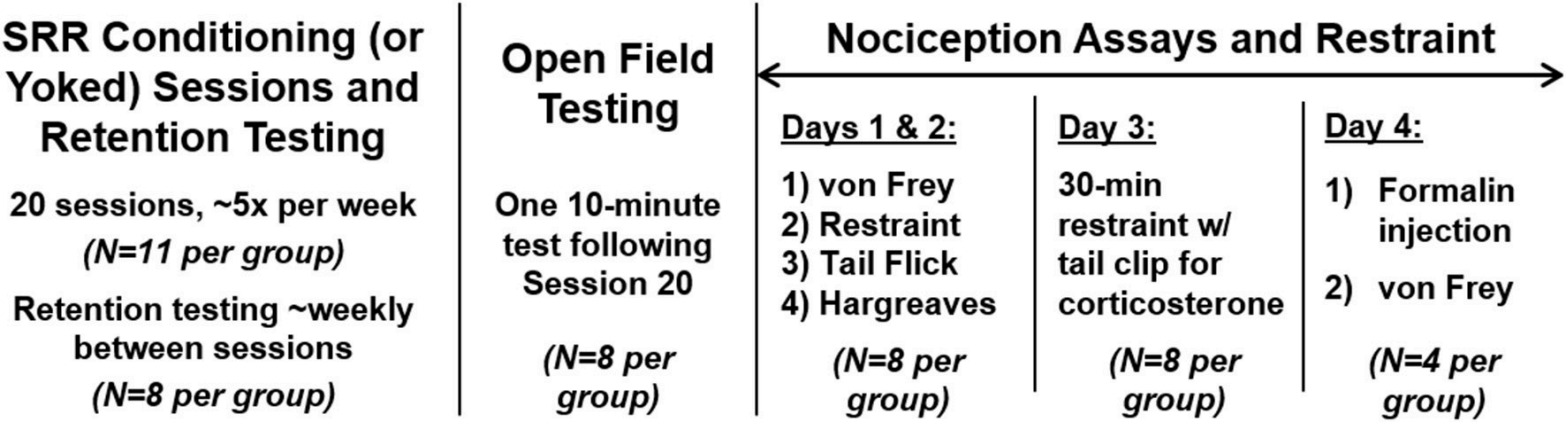
Timeline for experimental procedures. All animals underwent 20 sessions of SRR conditioning or paired yoked control procedures (*n* = 11 per group). A subset of rats (*n* = 8 per group) also underwent 3-5 SRR retention tests with no reinforcement and only background red light (“dark sessions”), intermittent with conditioning sessions. In this same subgroup, open field testing occurred following Session 20 of conditioning. Nociception assays then took place over 4 days, 5–6 days after conditioning (except for one cohort of *n* = 2 pairs that was delayed approximately a month and underwent several SRR reinstatement sessions beforehand); a subset of rats were injected with formalin on Day 4 to evaluate the development of mechanical allodynia in a von Frey Test conducted 75 min later (*n* = 4 per group). Acute restraint sessions and blood collection for plasma corticosterone assays were performed during the first 3 days of nociception assays. Not shown here are acclimation and nose poke sessions (described in Materials and methods). A separate subgroup of FRR conditioned rats and their yoked controls (*n* = 4 per group) underwent identical testing procedures to those shown here but received reinforcement for fast vs. slow RRs.

*Summary of conditioning procedures (see Materials and methods):* We continuously monitored RR using whole body plethysmography. Following acclimation (with the final 2 h considered as our “pre-conditioning” period), feedback-based conditioning of reduced RR was achieved using strobe light as negative reinforcement. Conditioning occurred over 20 2-h sessions, ~5 days per week. SRR conditioned rats turned off the strobe light when current RR ≤ target RR, while yoked control rats passively received the same stimulus (Figure 1). We also performed retention sessions to monitor persistence of reduced RR in the absence of reinforcement, as well as subsequent behavioral tests (Figure 2).

### Conditioned rats spent progressively more time breathing below the conditioned RR threshold of 80 breaths/minute

**Figure 3:**
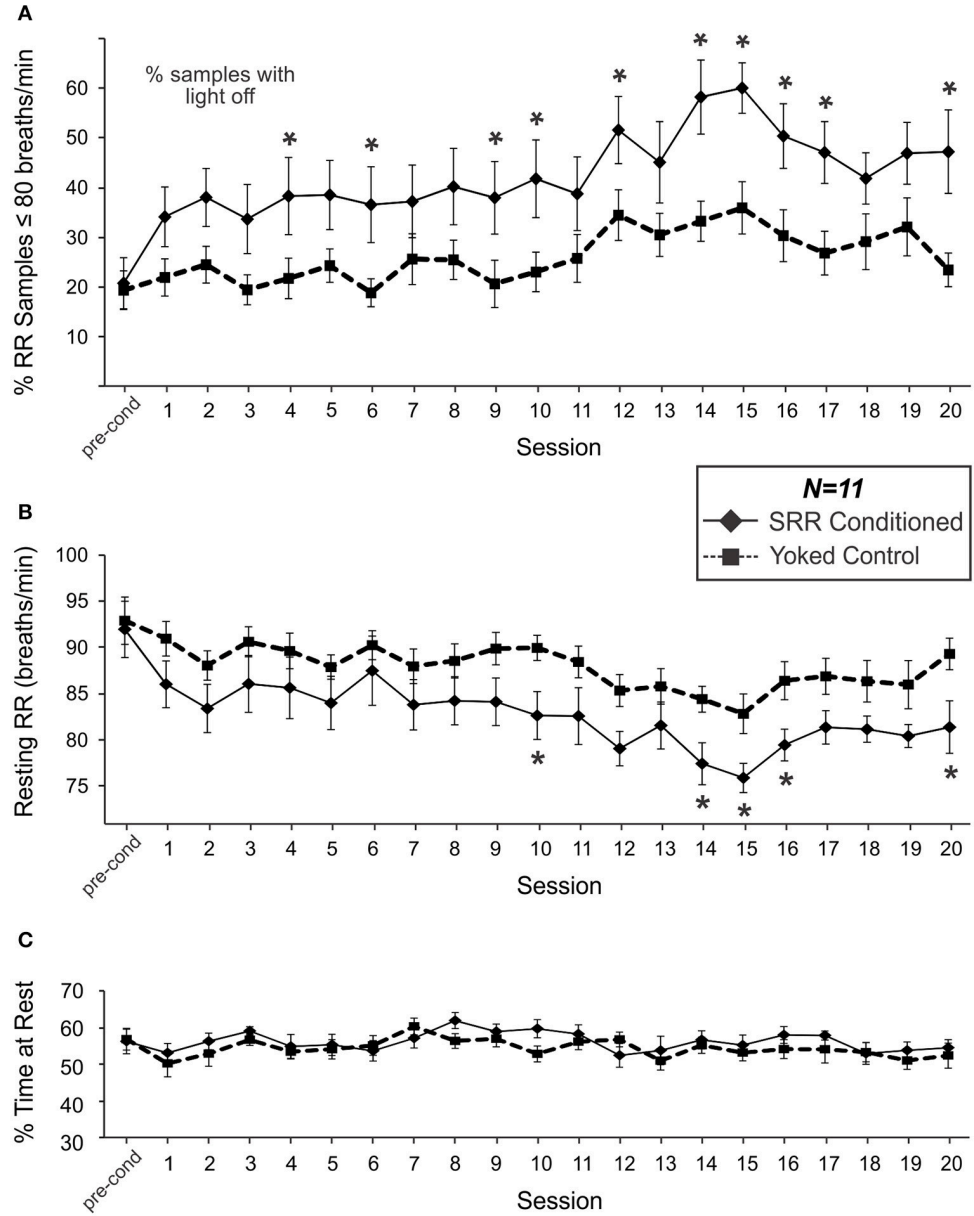
SRR conditioned rats learned to breathe more slowly and spent more time breathing below the conditioned RR threshold of 80 breaths/min. Group differences in respiration emerged over the course of 20 sessions. **(A)** The percentage of samples where RR met criterion (≤80 breaths/min). SRR conditioned rats displayed an increasing incidence of breaths below the target RR (*1-factor RM ANOVA*), and a greater % of samples under threshold than yoked controls (*2-factor RM ANOVA: significant main effect of Group*) over training. Multiple comparisons analysis revealed a significantly higher % of samples below threshold in SRR conditioned vs. yoked rats during Sessions 4, 6, 9, 10, 12, 14–17, and 20. **(B)** SRR conditioned rats decreased their average resting RR by 10.6 breaths/min (*1-factor RM ANOVA*) and their RR was lower than that of yoked controls (*2-factor RM ANOVA: significant main effect of Group)* over training. Follow-up testing revealed a significant difference between SRR conditioned and yoked animals in resting RR during Sessions 10, 14–16, and 20; conditioned rat resting RR values were >6 breaths/min lower than yoked control values in all cases. Note in **(A)** that % of samples under threshold for SRR conditioned rats is by definition % of samples in darkness, whereas for yoked rats the % of samples under SRR threshold did not have a direct relationship with the light being on or off. **(C)** SRR conditioned and yoked rats spent similar amounts of time resting. Average percentages of each 2 h session spent resting (% resting) for SRR conditioned rats and yoked controls are shown for pre-conditioning and Sessions 1–20. Animals typically spent between 50 and 60% of each trial at rest, with no differences observed between groups over the course of training. This suggests that factors other than differential activity levels were responsible for reducing resting RR in SRR conditioned rats. ^*^p < 0.05 *SRR conditioned vs. yoked, post-hoc Holm-Sidak comparisons*.

Figure 3A shows the percentage of samples where RR met the criterion value (RR ≤ 80 breaths/min) in SRR conditioned and yoked control animals over the training sessions. SRR conditioned rats displayed a significant increase in the incidence of breaths below the target RR of 80 breaths/min [*F*_(20, 200)_ = 4.38, *p* < 0.001; one-way RM ANOVA]. This was characterized by a sharp initial increase, followed by a more gradual increase over 20 conditioning sessions. The greatest inter-session % increase occurred between pre-conditioning and Session 1 (incidence increased from 20.8 to 34.2% of samples under threshold; a 64.4% increase), while incidence peaked at Session 15 (60.2% of samples under threshold; a 189% incidence increase from pre-conditioning). Overall, conditioned rats had a greater incidence of RR events below the preset light-off value than yoked rats [*F*_(1, 20)_ = 6.89, *p* = 0.016; 2-way RM ANOVA]. This effect was especially prominent during Session 14, when conditioned animals spent 58.4% of the session, on average, below the target RR vs. 33.4% for yoked controls. Still, yoked rats also experienced increased incidence of lower RR samples over sessions [$X_{\left(20,\;\text{N}\;=\;11\right)}^{2}$ = 40.0, *p* = 0.005; Friedman RM ANOVA on Ranks].

### Conditioned rats underwent significantly greater reductions in RR over the course of training

Mean resting RR for SRR conditioned rats during the pre-conditioning period was 92.0 breaths/min; this value was statistically indistinguishable from that established in a pilot study investigating the timeline of acclimation to the plethysmography setup, suggesting that all animals were acclimated prior to commencing conditioning procedures. SRR conditioned animals decreased their resting RR over 20 conditioning sessions [*F*_(20, 200)_ = 5.27, *p* < 0.001; one-way RM ANOVA; Figure 3B]. Effects were greatest during Session 15, with average RR decreasing by 17.5% (from 92.0 to 75.9 breaths/min). Yoked rats also showed a decrease in RR over sessions [*F*_(20, 200)_ = 2.75, *p* < 0.001; one-way RM ANOVA]. Nonetheless, SRR conditioned rats underwent significantly greater overall reductions in RR [*F*_(1, 20)_ = 4.70, *p* = 0.042; 2-way RM ANOVA]; this was most apparent during Sessions 14–16.

Interestingly, inter-session trends for both incidence of lower RR samples (RR ≤ 80 breaths/min) and average RR in yoked rats were broadly comparable in shape to those in SRR conditioned rats (Figures 3A,B). Indeed, individual RRs for SRR conditioned and yoked rats for each 2 h session were significantly correlated [*r*_(231)_ = 0.50, *p* < 0.001], as were values for % of samples under conditioning threshold [*r*_(231)_ = 0.49, *p* < 0.001]. We interpret this as an effect of shared experience, including shared housing and testing environments and identical exposure to visual stimulation during each conditioning session. For example, yoked rats may have found sessions with less light to be less stressful.

### % of time at rest

To assess whether SRR conditioning led to reductions in RR due to rats simply spending more time at rest—when RR would more likely be under training criterion e.g., (Guijt et al., 2007)—we compared % of time at rest between SRR conditioned rats and yoked controls. No differences in % of time spent resting were observed during pre-conditioning or any of the 20 training sessions [*F*_(1, 20)_ = 0.78, *p* = 0.39; 2-way RM ANOVA; power (α = 0.05) = 0.62; Figure 3C].

### The intra-session RR distribution for conditioned rats was shaped by the RR criterion and associated with reduced RR variability

**Figure 4:**
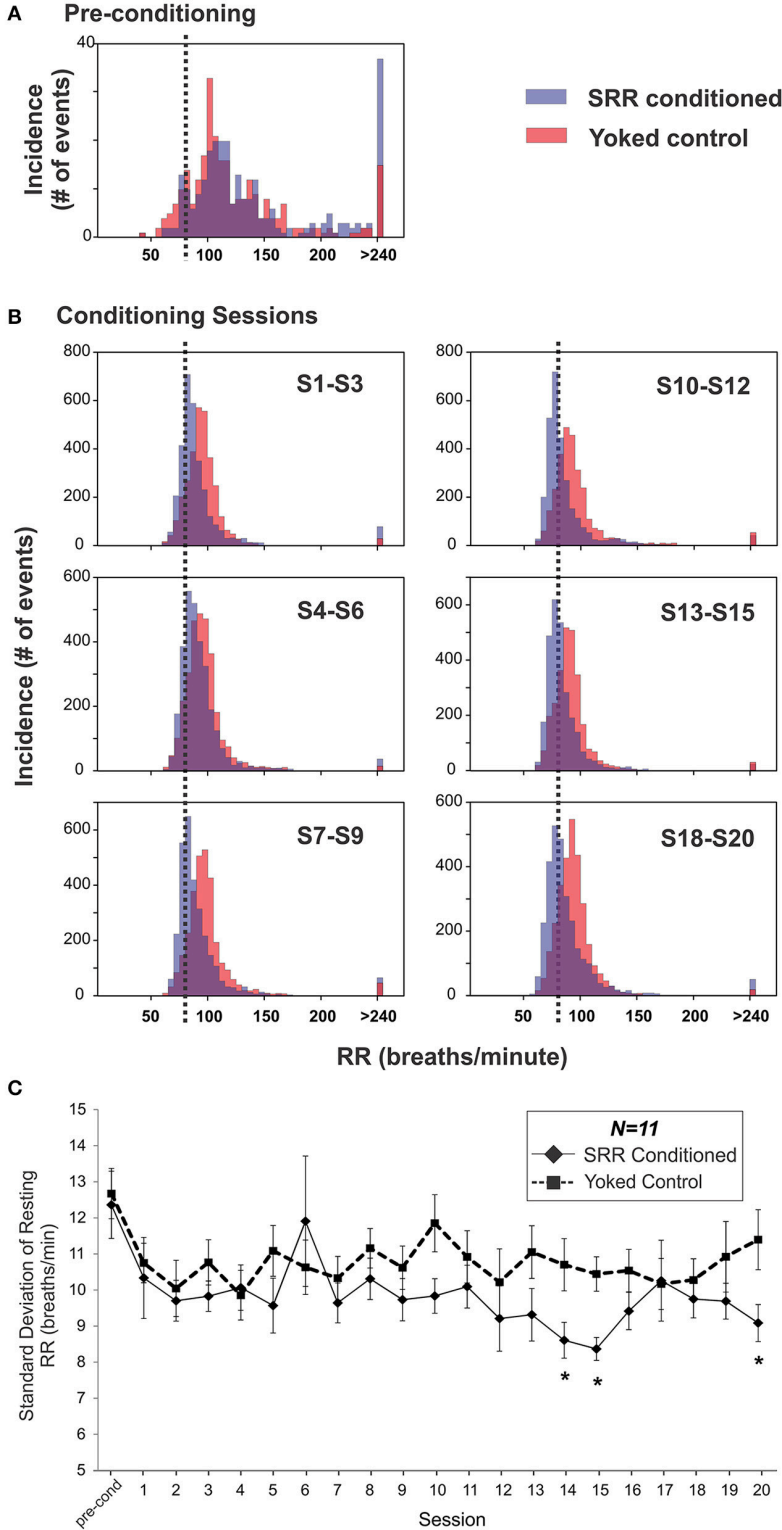
Respiration became slower and more regular over 20 sessions in SRR conditioned rats. **(A,B)** SRR conditioning led to a leftward shift in RR values and a skewed distribution centered just below the target RR. Shown are frequency histograms depicting the number of occurrences of different instantaneous RRs (5 breaths/min bins) over one 2 h pre-conditioning period or three conglomerated 2 h conditioning sessions in a representative pair of SRR conditioned (*blue*) and yoked control (*red*) animals. **(A)** Distributions during pre-conditioning. **(B)** Frequency histograms show the gradual leftward shift in RRs. Decreased rates (on the x-axis) are easily visualized in the conditioned rat, as is the increasing tendency to breathe just below the feedback threshold of 80 breaths/min (vertical black dotted lines). **(C)** Mean standard deviation of resting RR was used to assess reduction in RR variability. This index was reduced at Sessions 14, 15, and 20 in SRR conditioned vs. yoked rats, indicating more regular respiration. ^*^p < 0.05 *SRR conditioned vs. yoked, post-hoc Holm-Sidak comparisons*.

SRR conditioned and yoked rat RR distributions shifted toward lower values over the course of 20 training sessions. This is shown as a leftward shift from pre-conditioning in histogram plots, with separation between SRR conditioned and yoked animals being most evident in later sessions (Figures 4A,B). The shift in SRR conditioned rats was clearly controlled by the conditioning criterion (strobe off when RR ≤ 80 breaths/min), as there was a sharp increase in the incidence of values just below this criterion; this led to right-skewed distributions. A consequence of this skewed shift was decreased RR variability in SRR conditioned rats (Figure 4C). Mean RR variability, measured as standard deviation values, became lower in SRR conditioned rats over the course of 20 sessions [$X_{\left(20,\;\text{N}\;=\;11\right)}^{2}$ = 34.2, *p* = 0.025; Friedman RM ANOVA on Ranks]. SRR conditioned rats showed significantly increased regularity of breathing vs. yoked controls during Sessions 14, 15, and 20 (*p* < 0.05, *post-hoc* Holm-Sidak comparisons). Compared to pre-conditioning, the greatest reduction in variability in conditioned rats was seen in Session 15 (standard deviation decreased from 12.4 to 8.4 breaths/min; Figure 4C).

### SRR conditioned rats retained lower RRs between training sessions

**Figure 5:**
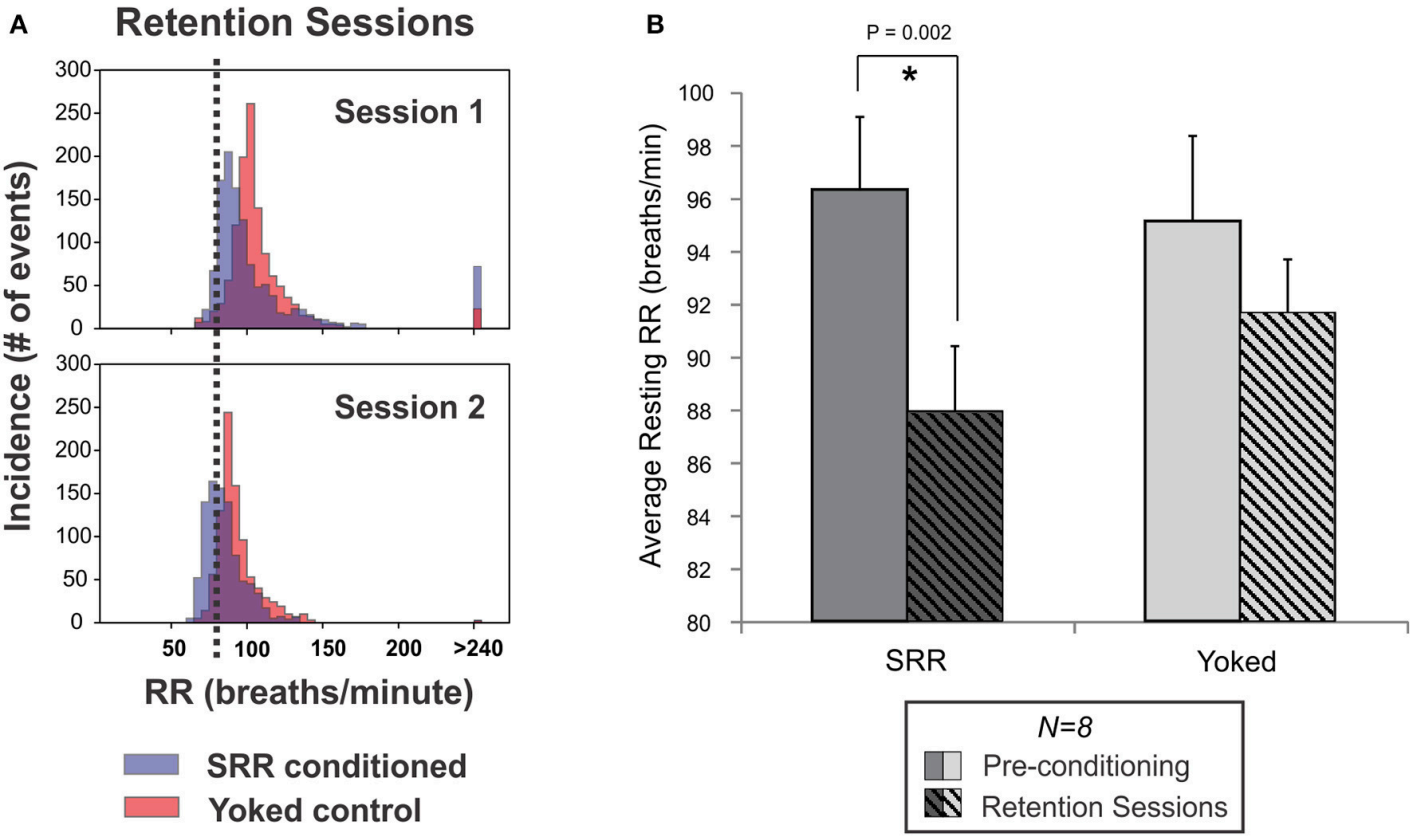
Reduced RR was retained between SRR conditioning sessions. **(A)** Individual frequency histograms for one pair of SRR conditioned (*blue*) and yoked control (*red*) rats during two representative retention sessions. These 2 h sessions were interspersed with normal conditioning and 4 days apart from one another (following Sessions 7 and 10, respectively). The RR threshold for conditioning (80 breaths/min) is shown with vertical black dotted lines. **(B)** Pooled results for all animals and retention sessions. SRR conditioned rats maintained trained reductions in RR during SRR retention sessions (^*^p < 0.05, *paired t-test*). Values averaged across SRR retention sessions were 87.9 ± 2.5 breaths/min for SRR conditioned rats (pre-conditioning: 96.3 ± 2.7 breaths/min) and 91.6 ± 2.0 breaths/min for yoked controls (pre-conditioning: 95.1 ± 3.2 breaths/min).

Retention of conditioned SRR was assessed by intermittently interposing 3–5 additional plethysmographic recording sessions that occurred in darkness (SRR retention sessions). These sessions took place throughout normal training in a subset of rats (*n* = 8 per group). Example RR frequency histograms for retention sessions are shown in Figure 5A. SRR conditioned rats retained reductions in RR by 8.4 breaths/min compared to pre-conditioning values [*t*_(7)_ = 4.79, *p* = 0.002; Figure 5B]. This was less than the RR reduction of 15.9 breaths/min obtained during SRR conditioning in the same animals. Interestingly, RR variability (standard deviation of resting RR) also decreased from pre-conditioning to retention sessions in SRR conditioned rats [*t*_(7)_ = 3.11, *p* = 0.017]. There was not adequate power to determine whether average RR and RR variability during retention sessions differed significantly between groups [average RR: power (α = 0.05) = 0.19; RR variability: power (α = 0.05) = 0.06]. Overall, these findings indicate a learned and potentially context-specific retention of reduced RR.

### RR up-conditioning supported learning effects in SRR conditioned rats

To exclude the possibility that reinforcer controllability was an essential variable contributing to the reduction of RR in SRR conditioned rats, we also undertook RR up-conditioning experiments [fast respiratory rate (FRR) conditioning; *n* = 4 pairs]. Mean pre-conditioning RR was similar between groups (89.5 and 86.2 breaths/min for conditioned and yoked rats, respectively). FRR conditioned rats breathed faster than their yoked controls throughout conditioning [*F*_(20, 120)_ = 1.89, *p* = 0.019, significant Group × Session interaction]. FRR conditioned rats had significantly higher RR values than yoked controls over the last two sessions of conditioning [average mean RR of 90.7 ± 1.1 vs. 78.4 ± 3.8 breaths per min; *t*_(6)_ = 2.62, *p* = 0.040], and displayed an overall trend toward increased RR from pre-conditioning to Session 20 [*F*_(20, 60)_ = 1.73, *p* = 0.054; power (α = 0.05) = 0.47]. Habituation leading to lower RR values may have precluded observations of an absolute increase in RR in FRR conditioned rats (e.g., yoked control rats had significantly reduced RRs over 20 sessions of SRR conditioning). Together, these results suggest that differential stimulus controllability did not account for RR reduction in SRR conditioned rats.

### RR and behavioral responses to experimental stressors associated with SRR conditioning

Following the training period, we undertook a variety of exploratory behavioral tests in the same subset of rats used for retention testing (*n* = 8 per group) to assess whether SRR conditioning associated with more chronic changes in RR and reduced sensitivity to experimental stressors.

#### Open field test of anxiety

**Figure 6:**
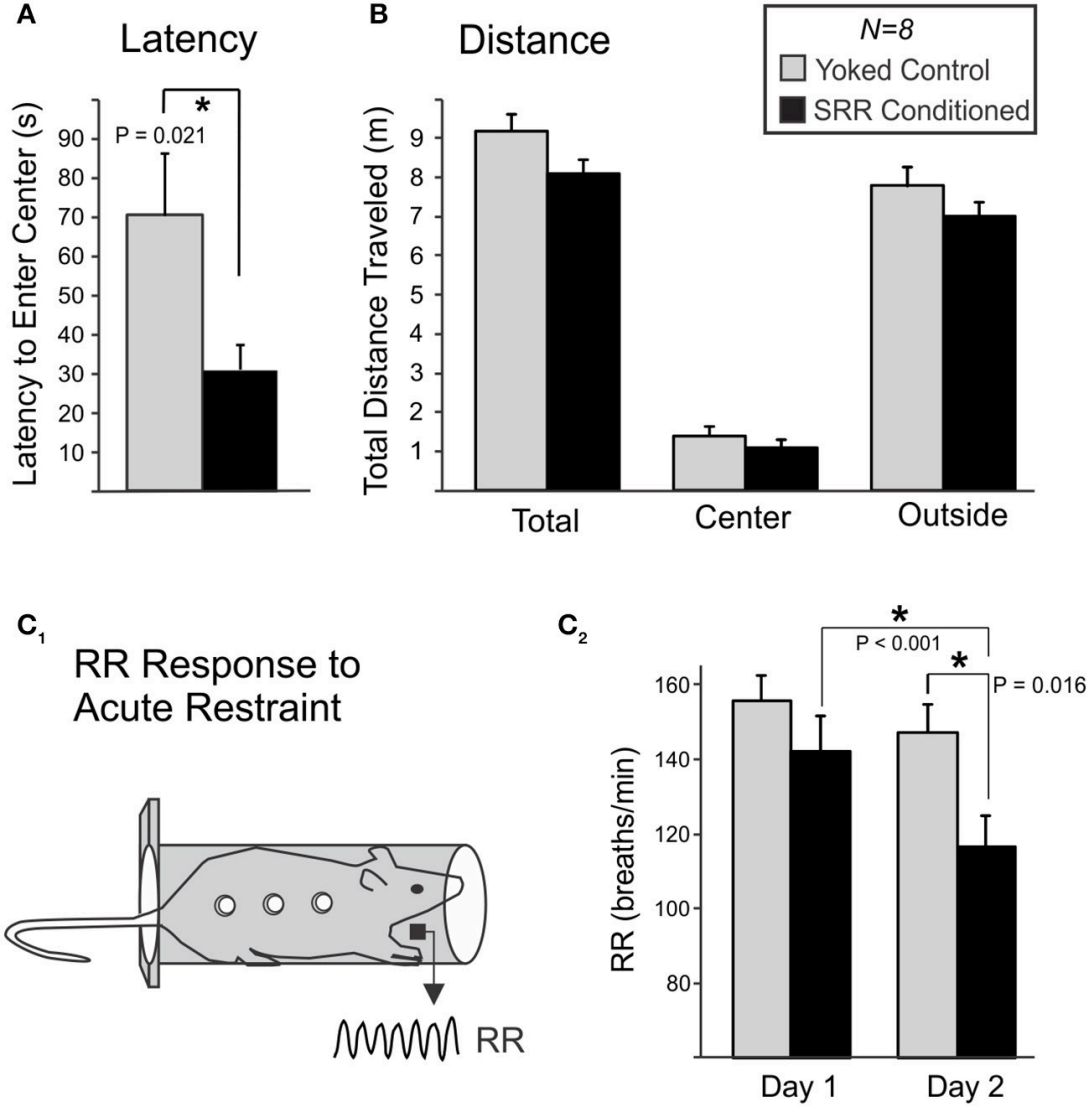
SRR conditioning reduced anxiety-like behavior in an open field and the respiratory response to acute restraint. Following 20 sessions of conditioning, rats were transferred to an open field and anxiety-like behavior was scored during 10-min sessions. **(A)** SRR conditioned rats showed a reduced latency to enter the center of the field vs. yoked controls (^*^p < 0.05, *Mann-Whitney Rank Sum test*). **(B)** SRR conditioned rats also showed a trend toward reduced distance traveled, in the arena as a whole (total) as well as in both defined areas (center and outside). **(C)** Rats later underwent 2 consecutive days of acute restraint. **(C**_1_**)** During these 10-min sessions, respiratory recordings were collected via electric field sensors positioned outside the restraint chamber. **(C**_2_**)** SRR conditioned rats had a lower RR response to restraint than yoked rats on the second day (^*^p < 0.05, *Student's t-test*), and decreased their RRs from Day 1 to Day 2 (^*^p < 0.05, *paired t-test*).

A 10 min Open Field Test assessed whether SRR conditioning reduced anxiety-like behavior immediately following the 20th training session (Figures 6A,B). SRR conditioned rats took less time than yoked controls to enter the center of the open field (31.1 ± 6.3 vs. 70.2 ± 16.0 s, respectively; Mann-Whitney *U* = 10.0, *n* = 8 rats per group, *p* = 0.021; Figure 6A). There was also a trend toward a smaller total horizontal distance traveled by SRR conditioned rats [SRR: 8.08 ± 0.36 m, Yoked: 9.20 ± 0.41 m; *t*_(14)_ = −2.05, *p* = 0.059; power (α = 0.05) = 0.48; Figure 6B]. Statistical tests for other conventional open field measures were underpowered [center field bouts: power (α = 0.05) = 0.087; duration in center: power (α = 0.05) = 0.16].

#### RR response to acute restraint

Ten-minutes restraint tests were conducted simultaneously with RR recordings to characterize the respiratory response to acute stress following SRR conditioning (Figure 6C). The mean RR response to restraint was significantly lower in SRR conditioned vs. yoked rats on the second of two consecutive days of testing [116.6 ± 8.2 vs. 147.2 ± 7.5 breaths/min, respectively; *t*_(14)_ = −2.74, *p* = 0.016]. Day 1 values were 142.2 ± 9.4 and 155.7 ± 6.7 breaths/min for SRR conditioned rats and yoked controls, respectively. Additionally, SRR conditioned rats decreased their RR by 25.6 breaths/min from Day 1 to Day 2 [*t*_(7)_ = 5.54, *p* < 0.001], while the decrease of 8.6 breaths/minute in yoked controls did not reach significance [*t*_(7)_ = 2.01, *p* = 0.084; power (α = 0.05) = 0.41].

#### Withdrawal reflex tests of nociception

**Figure 7:**
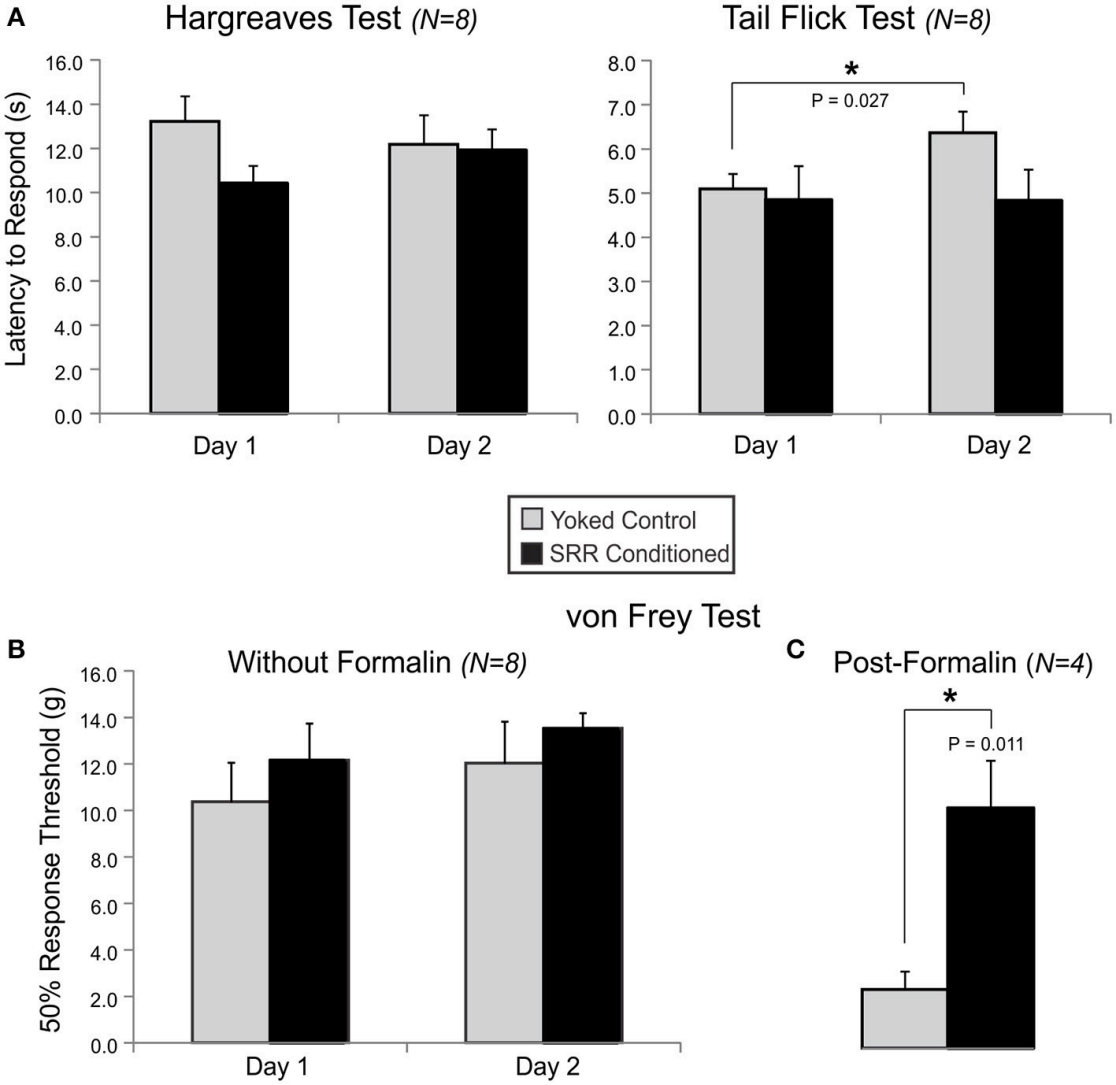
SRR conditioning prevented expression of mechanical allodynia following formalin injection. Shown are results for tests of thermal nociception in an open chamber (Hargreaves) or under restraint (Tail Flick), and mechanical nociception in an open chamber (von Frey) with or without prior formalin injection into the rat's right dorsal hindpaw. **(A)** There were no group differences in thermal sensitivity at baseline, despite SRR conditioned rats showing a trend toward increased responsiveness to the Hargreaves Test on Day 1 [10.4 ± 0.8 s vs. Yoked: 13.2 ± 1.1 s; *t*_(14)_ = −2.02, *p* = 0.064; power (α = 0.05) = 0.47] and Tail Flick Test on Day 2 [4.8 ± 0.7 s vs. Yoked: 6.4 ± 0.5 s; *t*_(14)_ = −1.81, *p* = 0.093; power (α = 0.05) = 0.39]. Yoked rats took more time to respond on Day 2 than Day 1 of the Tail Flick Test (^*^p < *0.05, paired t-test*). **(B)** Results from Days 1 and 2 on the mechanical von Frey Test did not clearly differentiate the two groups of rats. **(C)** Mechanical nociceptive responses to formalin injection were reduced in SRR conditioned vs. yoked rats on Day 4 of testing (^*^p < *0.05, Student's t-test*).

We performed a series of nociception assays (the von Frey, Tail Flick, Hargreaves, and formalin assays) to evaluate the impact of training on withdrawal responses to noxious stimulation. We did not observe differences between groups in withdrawal sensitivity to thermal (Figure 7A) or mechanical (Figure 7B) nociceptive stimuli at baseline, though yoked rats had reduced thermal sensitivity in the Tail Flick Test from Day 1 to Day 2 [*t*_(7)_ = −2.78, *p* = 0.027]. Although there were no group differences in hindpaw lifts during the 60-min period following formalin injection [*F*_(1, 6)_ = 0.004, *p* = 0.95; 2-way RM ANOVA], SRR conditioned rats displayed decreased mechanical sensitivity of the formalin-injected paw during a von Frey assay performed 75 min after injection [increased 50% response threshold; SRR: 10.2 ± 2.0 grams, Yoked: 2.5 ± 0.8 grams; *t*_(6)_ = 3.60, *p* = 0.011; Figure 7C].

## Discussion

We were successful in using operant conditioning procedures to reduce resting RR in adult rats. We used strobe light as negative reinforcement in an automated RR feedback paradigm triggered by real-time respiratory measurements provided via whole body plethysmography. Suppression of light was achieved when rats met a preset criterion of RR ≤ 80 breaths/min. Conditioning was implemented over 20 2 h sessions. Significant differences were seen by Session 4 with maximal effects occurring at Session 15—both in incidence of RR values meeting criterion and resting RR reduction. Although further work is needed to optimize the conditioning paradigm (e.g., providing fewer sessions) and more fully profile concomitant physio-behavioral changes, the present results are consistent with SRR being a physiologically controllable variable that may reduce responsivity to stressors.

### Factors relating to magnitude of RR reduction

At peak conditioning, SRR conditioned rats had 67.0% more samples under the feedback threshold (80 breaths/min) than yoked controls. SRR conditioned rats appeared to achieve this by maintaining RR just below criterion, as shown for an individual conditioned rat in Figure 4B. This coincided with a concomitant reduction in RR variability in SRR conditioned rats (Figure 4C), particularly during several later conditioning sessions. In assessing strength of conditioning, it is important to recognize that training was directly dependent on samples meeting criterion. The substantially greater % difference in samples meeting criterion (67.0%) than in average resting RR (8.4%) between groups at peak conditioning may thus be expected (Figures 3A,B). We also observed that reduced resting RR represented a learned event independent of broad changes in activity, as there were no differences in % time at rest between SRR conditioned and yoked rats (Figure 3C).

That average RR during pre-conditioning (92.0 breaths/min) was only marginally above the RR criterion for training (≤80 breaths/min) may have limited the magnitude of the changes observed. Conversely, setting the criterion below but near pre-conditioning RR increased the likelihood of the strobe light turning off to provide more opportunities for SRR conditioned rats to learn the response criterion, and this may have facilitated conditioning. As ours was the first study of its kind, it was essential to establish a robust paradigm rather than optimize conditioning by exploring a range of feedback-based approaches. Future studies attempting to extend this work may consider comparing results in groups having different target RRs, as well as progressively lowering RR criterion once conditioning has been established. Another possibility is applying a performance-dependent “moving” target RR, as used for operant conditioning of the H-reflex (Chen and Wolpaw, 1995). Also, as there is a reduction in RR during normal rat development (Reeves and Gozal, 2006; Huang and Goshgarian, 2009), greater magnitude RR reduction may have been achievable in younger rats; there may be age-dependent physiological constraints limiting the lower RRs an adult rat can sustain.

### Diminished efficacy of conditioning during sessions 17–20

The observation that RR conditioning peaked during Sessions 14–16 and then waned during the final few sessions suggests that repeated exposure to other training-related factors may have countered the retention of learning. Possible contributory factors include: (1) overexposure to the sensitizing strobe stimulus, (2) diminished aversive salience of the strobe, and (3) long-term disruption of circadian rhythms. (1) There is increased temporal lobe excitability in rats following exposure to repeated intermittent bright light (Uhlrich et al., 2005). In our study, continued strobe light conditioning may have generated a competing long-term sensitizing effect resulting in greater behavioral arousal by completion of Session 20. Such changes may explain the lack of significant differences in some open field variables and in CORT response to restraint. (2) Repeated strobe exposure could also have resulted in retinal deterioration and a corresponding loss of visual function (Gorn and Kuwabara, 1967; Hao et al., 2002), and therefore diminished the aversive salience of the negatively reinforcing strobe stimulus. We cannot rule out this possibility as we did not measure animal EEG or visual function/retinal atrophy. (3) A single exposure to bright light during the dark cycle can drastically alter rat melatonin levels and sleep cycles (Lewy et al., 1980). Thus, long-term strobe light exposure during the rat dark cycle could have disrupted circadian rhythms and progressively impaired retention of learning, but melatonin levels and home-cage activity were not quantified in our study.

### Predicted differences in tidal volume and possible metabolic changes due to a conditioned reduction in respiratory rate

One important consideration relates to a pivotal assumption we are making concerning slow breathing. The operant conditioning reinforced one respiratory parameter—reduced rate. It is assumed that slower breaths are also deeper, i.e., that minute ventilation remains constant. This is normally the case due to relatively stable animal metabolic demands, and is consistent with the observation that activity levels were similar between yoked and conditioned populations during training sessions. Nonetheless, it is important to acknowledge that our paradigm cannot rule out metabolic changes between groups as contributing to our observed results. Indeed, it is proposed that slowing respiration leads to enhanced ventilation-perfusion matching and possibly enhanced metabolic efficiency (Yasuma and Hayano, 2004; Anderson et al., 2009). Additionally, this study did not monitor arterial blood gases, so possible differences in PCO_2_, PO_2_, or pH between populations cannot be ruled out.

### Continued RR reduction during interspersed retention sessions and acute restraint suggesting long-term maintenance of learned slow breathing

To test for the maintenance of learned RR reductions in conditioned rats, additional sessions conducted without conditioning (retention sessions) were interspersed with conditioning sessions; these occurred 24–48 h after the previous conditioning session. Significant reductions in RR were retained during these sessions (Figure 5B) but were diminished in magnitude, suggesting partial behavioral extinction. Maintained RR reduction during retention sessions may have been attributable to context-specific expectation of conditioning (i.e., placement in the plethysmography chamber with expectation of light-based respiratory feedback). Additional studies are required to determine whether RR reductions are retained independent of behavioral context and expectation (e.g., continuous recordings of RR in an animal's home cage) (Noble et al., 2017). Whatever the mediating factors, our findings support retention of learned reductions in RR in the absence of conditioning.

Similar to fear conditioning studies that monitor the conditioned response in novel contexts (Maren et al., 2013), our investigation of the respiratory response to acute restraint can be viewed as an extension of our model to a novel context, independent of plethysmography, in order to evaluate persistence of the behavior. Acute restraint is also an established stressor (Heinrichs and Koob, 2006), and the extent of RR elevation during restraint stress has been demonstrated to correspond to an animal's anxiety state (Carnevali et al., 2013). It is important to emphasize a careful interpretation of our results given the use of reduced RR as our conditioned response. However, analysis of our restraint data supported both RR reduction in a novel context (i.e., SRR conditioned rats had lower RR compared to yoked controls), and reduced sensitivity to repeated priming of an experimentally validated stress response (i.e., magnitude of RR response to restraint was significantly reduced from Day 1 to Day 2 in SRR conditioned rats).

### Interpretation of open field latencies and distances traveled

Latency to enter the center of an open field is used as a measure of anxiety-like behavior (Prut and Belzung, 2003), with normal latencies ranging from 65 to 120 s in Sprague-Dawley rats (Nam et al., 2014). Observed performance in the yoked control population is consistent with this range (70 s). In contrast, SRR conditioned rats had a significantly reduced latency (31 s), indicative of decreased anxiety-like behavior in response to novelty stress.

An observed trend toward reduced horizontal distance traveled in conditioned rats (*p* = 0.059) could have obscured statistical detection of differences in other open field variables. This trend suggests that our conditioned animals were generally more restful following conditioning. This contrasts with the lack of difference in activity between SRR conditioned and yoked animals during conditioning in the plethysmography chambers, where motor activity was defined as the absence of rest and therefore included grooming behavior, exploratory sniffing, and vertical rearing. Additional studies are required to determine whether restful behavior is an important outcome of conditioning, and further, whether changes are seen across different behavioral contexts. For example, future studies could test for alterations in motor responses following a variety of stressors and incorporate tests specifically designed to quantify distinguishable categories of motor behaviors (Kalueff and Tuohimaa, 2005; Heinrichs and Koob, 2006).

### The impact of conditioned SRR on nociception and inflammatory pain

Long-term respiratory training had no observed effect on normal nociceptive reflex responses to thermal and mechanical stimuli. In comparison, mechanical withdrawal responses tested 75 min following formalin injection were significantly reduced. This supports a role for SRR in limiting inflammation and/or minimizing amplified signaling in neural circuits mediating allodynia. The fact that unprovoked behavioral responses (hindpaw lifts) to the formalin inflammatory pain model were indistinguishable between groups during the hour following injection argues for the latter possibility. For example, increased descending drive from modulatory systems that depress nociceptive pathways could lead to reduced responsivity to mechanical stimulation (Millan, 2002).

## Conclusions and future directions

Since ours is the first study to operantly condition SRR in an animal model and observe behavioral outcome, we are now in a position to link trained slowed breathing to physio-behavioral indices of stress sensitivity.

While we have not performed mechanistic studies to causally associate slow breathing with stress reduction, we did obtain some evidence of reduced sensitivity to experimental stressors in SRR conditioned rats. In the series of basic tests we performed in a subset of conditioned rats, latency to enter the center of an open field was reduced (Figure 6A) to well below the normal range reported (Nam et al., 2014), RR elevation was attenuated in response to restraint (Figure 6C_2_), and pain sensitization (allodynia) was absent following formalin-induced inflammation (Figure 7C). Decreased anxiety and stress reactivity are well-documented in meditation, yoga, and with paced breathing (McCaul et al., 1979; Sakakibara and Hayano, 1996; Brown and Gerbarg, 2005; Pace et al., 2009). The possibility that respiratory conditioning could limit injury-induced allodynia is intriguing as there is evidence that slowed respiration reduces pain sensitivity (Grant and Rainville, 2009; Zautra et al., 2010; Busch et al., 2012) and the perceived unpleasantness of pain is reduced in meditation (Zeidan et al., 2011; Lutz et al., 2013). Interestingly, the lack of an expected reduction in CORT levels following restraint in SRR conditioned rats is consistent with human meditation studies, which have found the cortisol response to acute stress to be unchanged or even greater in meditators despite decreased self-reported psychological stress reactivity (Pace et al., 2009; Creswell et al., 2014). Taken together, our findings provide preliminary evidence that trained slowed breathing can elicit a state of reduced stress responsiveness. However, more studies are needed to clarify the strengths and limitations of our model and to determine the mechanistic link between trained slowed breathing and reduced sensitivity to stressors. For example, recent studies in the mouse suggest that there are neuronal populations within the pre-Bötzinger complex that are specifically associated with slow breathing, and that they may have unique projections to circuits that are causal to subsequent observed changes in behavior (Yackle et al., 2017).

Alternative autonomic or immune markers could provide better metrics for the therapeutic impact of SRR. For instance, future studies could investigate sympathoadrenomedullary stress axis function (e.g., norepinephrine or epinephrine) or stress-induced inflammation (Raison et al., 2006) as indices of autonomic dysfunction. This is an especially important consideration due to our finding that SRR conditioned rats have an increased threshold of responsiveness to nociception during the chemical formalin assay (Figure 7C). Due to our desire to study freely behaving rats and avoid invasive telemetry procedures, we did not measure cardiac activity during the present study. Next generation operant conditioning studies will ideally monitor respiratory and heart rate using remote sensors to identify the impact of conditioning on cardiorespiratory synchronization, a key concomitant of slow breathing in humans (Lehrer et al., 1999). If rat autonomic function is already optimally tuned, such that SRR would not appreciably alter autonomic balance, we could precondition rats in an environment that is likely to promote stress. Many options are available (Heinrichs and Koob, 2006). Other possibilities include measurement of tidal volume (i.e., depth of ventilation) or use of methods that allow for quantification of metabolic changes. As slow respiration can entrain electroencephalographic (EEG) activity (Kamei et al., 2000; Busek and Kemlink, 2005), EEG recordings could be performed to determine changes in quantitative measures of arousal and sleep-wake cycles. Combining these measures with steps aimed at elucidating biological mechanisms, such as quantifying c-Fos expression in brain arousal centers including the amygdala, hypothalamus, and nucleus of the solitary tract (a key brainstem parasympathetic relay nucleus that receives extensive input from pulmonary afferents), or undertaking genetic approaches to selectively control populations of neurons, will help to determine the underlying basis of SRR's therapeutic benefits.

## Materials and methods

### Experimental animals

Adult male Sprague-Dawley rats (~90 days old at the start of experimental procedures) were housed in standard cages in a vivarium on a reverse 12:12-h light-dark cycle and were fed *ad libitum* standard rodent diets. All experiments were approved by the Animal Care and Use Committee of Emory University. The experiments conformed to national standards for the care and use of experimental animals and the American Physiological Society's “Guiding Principles in the Care and Use of Animals.”

### Light-based respiratory rate conditioning

#### Whole body plethysmography setup for recording respiration

We continuously monitored RR using whole body plethysmography. Respiratory measurements during a given session were collected similarly to those described previously (Wilkinson et al., 2010). Individual rats were placed in Plexiglas plethysmography chambers (PLY3215, Buxco Research Systems), and respiration was measured using continuous flow barometric plethysmography (Jacky, 1980). A flowmeter (EW-32013-13, Cole-Parmer) was set to provide compressed air at a constant rate of 3 l/min, while PE-20 tubing filling the top 7 cm of the inflow line provided high input impedance. Air exited the chamber via a vacuum valve (SS-4MG, Nupro) connected to a vacuum pump. We adjusted the vacuum valve (while keeping the inflow set to 3 l/min) in small increments as necessary to maintain a chamber pressure near atmospheric level, as monitored via water manometer. Pressures remained relatively stable and adjustments were rarely necessary once a given 2 h conditioning session had commenced. Pressure changes were measured using a differential pressure transducer referenced to atmosphere (DP45, Validyne); output from a connected carrier demodulator (CD15, Validyne) was sent to a digital data acquisition system (PCI-6221 multifunction DAQ board, National Instruments). Analog signals were digitized at unity gain and a sample rate of 1–10 kHz. The digitized data was continuously output to a Windows computer running LabVIEW (National Instruments) to provide an accessible voltage readout. Prior to each experiment, calibration air pulses ranging from 0.5 to 2 ml were injected into the chamber using a syringe, to confirm signal fidelity.

#### LabVIEW feedback program and behavioral conditioning paradigm

Intermittent bright light (8 Hz) was used as negative reinforcement for conditioning reduced RR (Figure 1). Previous studies have shown that bright light maintains aversive salience for at least 60 h in albino rats (Barker et al., 2010), and intermittent bright light (strobe) is a common component of chronic mild stress paradigms in the rodent (Willner et al., 1987; Forbes et al., 1996; Bortolato et al., 2007). The preset criterion to turn off the strobe was RR ≤ 80 breaths/min. The specialty built setup for visual reinforcement consisted of two LED array panels projecting from above and below the plethysmography chamber, in order to prevent animals from burrowing to escape the stimulus (Barker et al., 2010). When the strobe light was on, light intensity at the chamber floor was 8,073 lux, measured with a digital light meter (401027, Extech Instruments). A red LED bulb provided constant background lighting largely outside of the rat visual spectrum (Burn, 2008). Because LEDs do not produce heat in the form of infrared radiation, there was negligible increase in the surrounding temperature. Due to our desire to follow a non-invasive protocol, we did not measure animal body temperatures during experimentation. Ambient chamber temperature was measured at several time points and remained relatively constant.

Recorded data were processed by a customized software interface in LabVIEW to monitor respiration and provide LED feedback concurrent with plethysmographic recordings. There were a number of controllable parameters. We preset the interface to sample within the known physiological range of respiratory frequencies (filter bandwidth of 1–5 Hz), with 10 breaths captured in a sample block. The Buneman Frequency Estimator subroutine was called to find the dominant peak, with the output being RR in breaths/min. The Buneman estimate is better than the traditional fast Fourier transform (FFT) because it will interpolate the peak in the frequency spectrum, even if it is between discrete points on the spectrum graph. For conditioning, real-time RR was compared to a user-defined target RR by a controller that triggered activation of the LED panels when recorded RR exceeded the preset value of 80 breaths/min. Animals thereby experienced real-time variation in presentation of the light stimulus (on or off) corresponding to their RR, representing a closed-loop feedback control system for conditioning of RR.

Once parameters were set for a given conditioning session, the program was started and allowed to run for 2 h. Simultaneously, video was captured (V-UCC22, Logitech) to permit real-time tracking and *post-hoc* confirmation of animal activity status. Throughout the session, animal activity levels and RRs were monitored and compared to voltage readouts in the interface by an observer following the animals remotely in an adjacent room. Upon session termination, raw voltage traces were automatically saved for subsequent analysis with pCLAMP analysis software (Molecular Devices). The corresponding video file for the trial was also saved. For each session, the percentage of samples meeting criterion (i.e., % of samples where RR ≤ 80 breaths/min) was calculated to assess the efficacy of conditioning.

### Assignment of experimental groups—SRR conditioned rats vs. yoked controls

Prior to acclimation of experimental animals, paired littermate rats were randomly assigned as “SRR conditioned” or “yoked control”. A yoked control design matches animals in pairs such that they experience similar environmental conditions and undergo identical stimulation; however, only the experimental animal undergoes a specific manipulation of interest (association of visual stimulation with its respiration, i.e., operant conditioning of reduced RR). SRR conditioned rats were able to turn off the strobe light when current RR ≤ target RR, while yoked control rats passively received the same stimulus (Figure 1). An opaque barrier prevented animals from seeing each other, while the vacuum used for air outflow provided constant background noise to prevent auditory communication during experimental procedures (although ultrasonic vocalizations could not conclusively be ruled out). In order to prevent baseline data biases and screen for rats able to learn an operant task, we preset exclusion criteria for respiratory conditioning. Experimental pairs were excluded if pre-conditioning differences in RR were >20 breaths/min or if animals failed to learn a nose poke task (see below).

#### Acclimation to plethysmographic chambers and nose poke sessions for trainability

Based on results from plethysmographic recordings in a separate subgroup of animals, we acclimated all animals to be used for conditioning studies to the dual-chamber plethysmography setup for 12 h over 3–6 sessions prior to commencement of experimental procedures. Respiratory recordings from the final 2 h of acclimation (once RR had stabilized) were used for analysis as our “pre-conditioning” session. 1–2 days following the final acclimation session, paired animals were once again transferred to plethysmography chambers, and conditioned rats were trained in a simple nose poke task to establish animal trainability. This task lasted for 30 min, and was repeated over 3 days. During these sessions, the strobe light turned off whenever the animal poked its nose into a small aperture with an infrared beam designed to detect each poke. All conditioned rats that performed ≥20 nose pokes per session by the 3rd 30 min session, and their yoked controls, were included in the study. These sessions also had the effect of acclimating animals to all experimental conditions (including differential stimulus controllability) except for RR-based conditioning.

### Experimental timeline

The experimental timeline for SRR conditioning sessions, SRR retention testing, and subsequent behavioral tests is outlined in Figure 2 and described in detail below.

#### SRR conditioning sessions

Feedback-based conditioning of reduced RR was accomplished using strobe light as a chronic mild stressor for negative reinforcement. Conditioning occurred over the course of 20 2-h sessions, ~5 days per week (*n* = 11 pairs of SRR conditioned and yoked control rats). Conditioning schedules varied slightly between animal pairs as we attempted to maximize strobe's efficacy as a negative reinforcer and mimic chronic mild stress paradigms by increasing the unpredictability of our reinforcement schedule (Willner et al., 1987).

#### Determination of % of samples meeting criterion, average resting RR, and % of time spent resting

Dependent variables measured were average RR (breaths/min) and the percentage of samples meeting criterion (the percentage of captured epochs, each 10 breaths long, where current RR ≤ target RR). Although % of samples meeting criterion provided a direct measure to assess the success of our training paradigm, it did not address the key question of whether operant procedures resulted in a trained reduction in resting RR over time. This question is important since slowed baseline respiration may trigger a number of therapeutic benefits associated with reduced reactivity to stressors. Therefore, we also measured resting RR.

Raw plethysmographic recordings output from LabVIEW provided adequate information to determine an animal's average resting RR and % of trial at rest. To calculate these variables for each session, we detected individual breaths and removed movement-related values to isolate periods of rest. All analysis was performed in Clampfit (pCLAMP analysis software, Molecular Devices) following 5 Hz low-pass Gaussian filtering. We performed threshold-based analysis to obtain instantaneous RRs over individual 2 h trials. We then generated scatterplots of instantaneous RR, excluding the first 20 min (acclimation period) and periods of movement. From the remaining epochs, the average resting RR (± standard deviation) was calculated over the course of ~100 min periods. To determine % of time at rest (vs. moving), the calculation was: (1/average resting RR in Hz) × (number of resting breaths) / (total session time in seconds) × 100.

#### SRR retention testing

We hypothesized that RRs following SRR training would be lower than pre-conditioning values, so undertook intermittent retention sessions in a subset of rats (*n* = 8 per group, also used for experimental stress tests described below). This entailed running approximately weekly 2 h respiratory recording sessions in the absence of visual reinforcement (“SRR Retention Sessions”). Each of these sessions occurred 24–48 h after a previous SRR conditioning session. Retention sessions began after successful acquisition of respiratory learning (i.e., after Session 4; see Figure 3A). If rats retained a persistently lower baseline RR, this could in turn mediate any differences observed during open field testing or subsequent outcome measures.

#### Up-conditioning control group

Since the degree of control an animal has over environmental influences can impact behavioral and autonomic functioning (Maier, 1986; Burgess et al., 1993), we established an additional control population to exclude the possibility that reinforcer controllability was an essential variable contributing to the reduction of RR in SRR conditioned rats. While learned helplessness may engage the sympathetic nervous system (Peters et al., 1998), control over one's environment is known to be therapeutic (Rodin and Langer, 1977). Therefore, because yoked rats lacked control over experimental reinforcement, one might have expected their RRs to gradually increase relative to those of SRR conditioned rats simply due to the impact of learned helplessness on sympathetic arousal. Inversely, the progressive decrease in RR in SRR conditioned rats might be explained not by operant learning, but by the therapeutic impact of stimulus controllability. To rule out this confound, a separate subgroup of rats was conditioned to increase their RR (fast respiratory rate [FRR] conditioned; *n* = 4). These rats were assigned to receive “up-conditioning” for fast (instead of slow) breaths. Procedures were identical to those for SRR training (described above), except that high RRs were reinforced, so that the aversive strobe light turned off when current RR ≥ 90 breaths/min (*n* = 3 rats) or 80 breaths/min (*n* = 1 rat); in the former case, the threshold was decreased to 80 breaths/min for two rats during early conditioning to prevent potential strobe overexposure. Following identical acclimation and nose poke procedures as for SRR conditioning, paired FRR conditioned rats and yoked controls underwent 20 sessions of RR up-conditioning.

#### Experimental stressors

##### Open field test of anxiety

Following Session 20 of SRR conditioning, rats were transferred to a large open field apparatus and allowed to freely explore the environment for 10 min while being recorded over video. The Open Field Test is commonly used to monitor rodent exploratory and anxiety-like behavior in a large novel environment (Broadhurst, 1957; Ennaceur et al., 2006). CleverSys TopScan software was used to analyze the corresponding video files. Center was defined as the inner 50% area of the surface. Horizontal distance traveled, number of center field bouts (entries and exists), duration of the trial spent in the center of the arena, and latency to enter the center of the arena were measured over the course of the 10-min session.

##### Nociception assays and acute restraint stress

Approximately one week after conditioning (5–6 days following Session 20, except for one cohort of *n* = 2 pairs that was delayed to 34–42 days post-training; reinstatement sessions were conducted at several time points in this cohort to ensure maintenance of the conditioned response), rats were transferred to a novel behavioral testing room and acclimated in small acrylic chambers for 30 min. Subsequently, a series of nociception and stress assays were performed over the course of 4 days:

*Days 1 and 2:* Following the 30 min acclimation period, individual animals were tested using the mechano-nociceptive von Frey Test, during which calibrated von Frey hairs (NC12775-99, North Coast Medical, Inc.) were used to test each rat's sensitivity to mechanical stimulation of the hindpaw. Procedures and analysis were similar to existing methods (Chaplan et al., 1994). After completing von Frey testing in both animals, they were sequentially moved to a small restraining cylindrical tube (Rodent Restrainer, IITC Life Science) for a 10-min acute restraint test. During this test, electric field sensors (EPIC, Plessey Semiconductors) were affixed to the side of the tube to record RR during restraint (see Noble et al., 2017). Immediately following restraint, rats remained in the same tube for another 10 min for the thermo-nociceptive Tail Flick Test (Tail Flick Analgesia Meter, IITC Life Science), which involved application of radiant heat aimed at a rodent's tail to induce a withdrawal response. The heat turned off automatically when the tail was withdrawn or a maximal cutoff of 8 s was reached. Animals were tested three times, each 2 min apart, and the last two were averaged to obtain withdrawal latencies. Immediately following 20 min in the cylindrical chamber (pre-Tail Flick restraint test and Tail Flick Test) for both animals, paired rats were moved to acrylic chambers identical to those used for von Frey testing and our second thermo-nociceptive assay, the Hargreaves Test, was performed (Plantar Test Apparatus, IITC Life Science). This test also used radiant heat to induce withdrawal, but in this case the stimulation was directed at the hindpaw rather than at the tail. Right and left paws were each tested 3–5 times, with at least 5 min between replicate measurements, and the closest two values were averaged to obtain withdrawal latencies. Notably, the von Frey and Hargreaves Tests were performed in an open chamber, while the Tail Flick Test was undertaken with rats restrained in a conical Plexiglas cylinder.

*Day 3:* A 30-min acclimation period was followed by 30 min of restraint with subsequent tail clip to obtain blood samples for corticosterone (CORT) assays (see below).

*Day 4:* A subset of the rats undergoing nociception testing (*n* = 4 per group) were again acclimated for 30 min, and then immediately underwent the formalin assay in an attempt to induce allodynia (a painful response to innocuous stimulation indicative of central sensitization). An experimenter injected a 50 μl solution of 5% formalin into the dorsal surface of the animal's right hindpaw. The rats were observed for 90 min, during which they exhibited stereotypical behaviors such as lifting and licking the affected paw. As a conventional measure for the formalin assay, hindpaw lifts were scored in 5-min bins over the first 60 min of testing. Importantly, the formalin assay produces a response in two discrete phases, with the early phase reflecting activation of peripheral nociceptors, and the later one reflecting central sensitization and peripheral inflammatory mechanisms (Tjolsen et al., 1992). In addition to monitoring formalin-induced hindpaw lifts, animal responsiveness to mechanical stimulation was tested using a classical assay for allodynia (von Frey hairs) 75–90 min following injection to assess modulation of inflammatory pain (Garraway et al., 2009).

##### Corticosterone assays

The 30-min restraint session interspersed with nociception assays was longer than those monitoring the RR response to acute stress in order to allow sufficient timing for CORT elevation. Following restraint, blood samples were collected within 3 min of tail clip. Once collected, samples were processed for blood plasma. Plasma samples were diluted 1:40 and CORT levels were assayed with a commercially available ELISA kit (ADI-900-097, Enzo Life Sciences) in duplicate (sensitivity: 27.0 pg/ml; intra-assay coefficient of variation: 3.0%), in order to investigate the neuroendocrine response to acute stress following conditioning.

### Statistical analysis

#### Respiratory conditioning

Data are presented as mean ± SEM, unless otherwise noted. % of RR samples under threshold, average resting RR, standard deviation of resting RR, and % of trial at rest were analyzed with repeated measures (RM) ANOVAs with session as a within-subjects factor, and group (conditioned vs. yoked) as a between-subjects factor when 2-way RM ANOVAs were used. *Post-hoc* tests (Holm-Sidak method) corrected for multiple comparisons were performed in the case of significant results. When Normality or Equal Variance tests failed, the Friedman RM ANOVA on Ranks was performed as a one-way ANOVA alternative. Although the Shapiro-Wilk test indicated non-normality of the data for % of samples under threshold in SRR conditioned vs. yoked rats, linear regression analysis (a non-parametric substitute for two-factor ANOVAs) confirmed all significant results reported here. Despite the inherent advantages of a yoked design (control over all extraneous variables other than stimulus controllability), independent (vs. paired) *t*-tests were used to compare groups since paired tests required non-validated assumptions about the amount of variability between experimental subjects (paired rats) at baseline. For retention sessions, average resting RR and standard deviation of resting RR did not depend on when these sessions were run, so values were averaged and compared to pre-conditioning values within a given group using paired *t*-tests; SRR conditioned vs. yoked rat retention session comparisons were made using Student's *t*-tests.

#### Experimental stressors

For open field analysis, variables (including bouts and duration in center, and distance traveled) were scored across the 10-min period, and planned Student's *t*-tests were performed between groups (SRR conditioned rats vs. yoked controls), with individual variables analyzed separately (e.g., see Smith et al., 2007). Latency to enter the center of the open field and CORT response to restraint were analyzed using the non-parametric Mann-Whitney Rank Sum Test, as data failed to reach normality. Nociception assay responses and acute restraint RRs were analyzed with planned Student's *t*-tests when comparing SRR conditioned rats and yoked controls, and paired *t*-tests when comparing between days within a given group. Hindpaw lifts in the formalin assay were quantified in individual 5-min epochs throughout the first 60 min of the test and analyzed using a 2-way RM ANOVA, with time (12 consecutive epochs) as the within-subjects factor and group (conditioned vs. yoked) as the between-subjects factor. Statistics were performed with SigmaPlot with significance set at *p* < 0.05 and two-tailed tests. No prior studies existed to provide information for appropriate sample size determination; our own pilot research suggested that sample sizes of *n* = 5–24 rats per group (SRR conditioned and yoked control) would provide adequate power (0.80) to detect significant group differences in RR during conditioning. Unfortunately, a number of statistical tests related to our behavioral assays were not adequately powered to detect significant differences (see Supplementary Table 1). Statistical tests for conventional open field measures of center field bouts and duration of time spent in the center, withdrawal responses to basic nociceptive stimuli (Tail Flick Test Day 1 and Hargreaves Test Day 2), SRR conditioned vs. yoked rat RR during retention sessions and in response to restraint on Day 1, and corticosterone levels in response to restraint were all underpowered (power with α = 0.05 < 0.30).

## Ethics statement

This study was carried out in accordance with the recommendations of the American Physiological Society's “Guiding Principles in the Care and Use of Animals.” The protocol was approved by the Animal Care and Use Committee of Emory University.

## Author contributions

DN and SH were responsible for experimental design/execution and wrote the majority of the finished manuscript. WG designed the LabVIEW feedback program and contributed extensively to experimental methods. SG and KM ran nociception assays and helped with data analysis and interpretation. All authors participated in manuscript edits and approved the final version.

### Conflict of interest statement

The authors declare that the research was conducted in the absence of any commercial or financial relationships that could be construed as a potential conflict of interest.
