# Supplementary material for: Slow Breathing Can Be Operantly Conditioned in the Rat and May Reduce Sensitivity to Experimental Stressors
Source: Front Physiol. 2017 Oct 30;8:854. doi: 10.3389/fphys.2017.00854 (PMC5670354; doi:10.3389/fphys.2017.00854)
Supplement: Supplementary file 1 [file Table1.PDF]

Key for Table of Power Values

Clearly adequate power (> 0.80)	Borderline adequate power (> 0.50)
Relatively low power (> 0.30)	Inadequate power = EXCLUDED (< 0.30)
N.S. = Not significant (p > 0.10)	Excluded from manuscript
S = Statistically significant (p < 0.05)	~S = Borderline significant (p < 0.10)

Table I. Power Values for Outcome Measures (in order of power, high → low)

Statistical Test	Reported Result	Power of performed test with alpha = 0.05
SRR Restraint Day 1 vs. 2 (paired t-test)	S	0.997
SRR Retention vs. Pre-conditioning RR (paired t-test)	S	0.982
Formalin von Frey (t-test)	S	0.849
SRR STDEV Retention vs. Pre-cond. RR (paired t-test)	S	0.759
Restraint Day 2 (t-test)	S	0.723
Yoked Tail Flick Day 1 vs. 2 (paired t-test)	S	0.665
Open Field Latency (Mann-Whitney Rank Sum Test)	S	0.57 (for t-test)
Open Field Distance (t-test)	~S	0.480
Hargreaves Day 1 (t-test)	~S	0.467
Yoked Restraint Day 1 vs. 2 (paired t-test)	~S	0.412
Tail Flick Day 2 (t-test)	~S	0.391
Yoked STDEV Retention vs. Pre-cond. RR (paired t-test)	N.S.	0.198
SRR vs. Yoked Retention Session RR (t-test)	N.S.	0.194
Restraint Day 1 (t-test)	N.S.	0.193
Open Field Center Duration (t-test)	N.S.	0.157
Yoked Retention vs. Pre-conditioning RR (paired t-test)	N.S.	0.152
Open Field Center Bouts (t-test)	N.S.	0.087
CORT (Mann-Whitney Rank Sum Test)	N.S.	0.07 (for t-test)
Tail Flick Day 1 (t-test)	N.S.	0.059
SRR vs. Yoked STDEV Retention Session RR (t-test)	N.S.	0.058
Hargreaves Day 2 (t-test)	N.S.	0.053
SRR Tail Flick Day 1 vs. 2 (Wilcoxon Signed Rank Test)	N.S.	0.05 (for t-test)
